# Altered Spontaneous Brain Activity Patterns of Meibomian Gland Dysfunction in Severely Obese Population Measured Using the Fractional Amplitude of Low-Frequency Fluctuations

**DOI:** 10.3389/fpsyt.2022.914039

**Published:** 2022-05-11

**Authors:** Yu-Ling Xu, Xiao-Yu Wang, Jun Chen, Min Kang, Yi-Xin Wang, Li-Juan Zhang, Hui-Ye Shu, Xu-Lin Liao, Jie Zou, Hong Wei, Qian Ling, Yi Shao

**Affiliations:** ^1^Department of Ophthalmology, The First Affiliated Hospital of Nanchang University, Nanchang, China; ^2^Department of Ophthalmology and Visual Sciences, Cardiff University, Cardiff, United Kingdom; ^3^Department of Ophthalmology and Visual Sciences, The Chinese University of Hong Kong, Shatin, Hong Kong SAR, China

**Keywords:** severe obesity, meibomian gland dysfunction, fALFF, RS-fMRI, spontaneous brain activity

## Abstract

**Objective:**

Utilizing the fractional amplitude of low-frequency fluctuations (fALFF) technique, this study sought to correlate spontaneous cerebral abnormalities with the clinical manifestations of meibomian gland dysfunction (MGD) in severely obese (SO) population.

**Subjects and Methods:**

Twelve MGD patients in SO population (PATs) (4 males and 8 females) and twelve healthy controls (HCs) (6 males and 6 females) matched by gender and age were enrolled. Every participant underwent resting-state functional magnetic resonance imaging (rs-MRI) scanning. Spontaneous cerebral activity alterations were examined using the fALFF method. Receiver operating characteristic (ROC) curves were utilized to classify the medial fALFF values of the PATs and HCs. PATs were also asked to complete anxiety and depression score forms, permitting a correlation analysis.

**Results:**

In contrast with HCs, PATs had prominently increased fALFF values in the left lingual gyrus, the right globus pallidus, the right anterior cingulate and paracingulate gyri and the left middle occipital lobe (*P* < 0.05), and decreased fALFF values in the right cerebellum, the left fusiform gyrus, the right medial orbitofrontal gyrus, the left triangle inferior frontal gyrus and the left inferior parietal gyrus (*P* < 0.05). The results of the ROC curve indicated that changes in regional fALFF values might help diagnose MGD in SO population. Moreover, fALFF values in the right cerebellum of PATs were positively correlated with hospital anxiety and depression scores (HADS) (r = 0.723, *P* = 0.008). The fALFF values in the left triangle inferior frontal gyrus of PAT were negatively correlated with HADS (r = −0.651, *P* = 0.022).

**Conclusions:**

Aberrant spontaneous activity was observed in multiple regions of the cerebrum, offering helpful information about the pathology of MGD in SO population. Aberrant fALFF values in these regions likely relates to the latent pathologic mechanisms of anomalous cerebral activities in PATs.

## Introduction

Obesity is an epidemic that affects 2 billion adults and 42 million children worldwide. Complications of obesity, such as hypertension and type 2 diabetes, are increasing (1). Obesity increases the risk of chronic organ failure, metabolic disease, and cancer. Further, it also increases the incidence of complications from acute illness (2). BMI (in kg/m^2^) is the basic unit for most of the epidemiologic data about obesity, using the range of 18.5–24.9 to define normality, 25.0–29.9 for rank I obesity, 30.0–34.9 for rank II obesity, 35.0–39.9 for rank III obesity and ≥ 40.0 for rank IV or morbid obesity (3).

**Figure 1:**
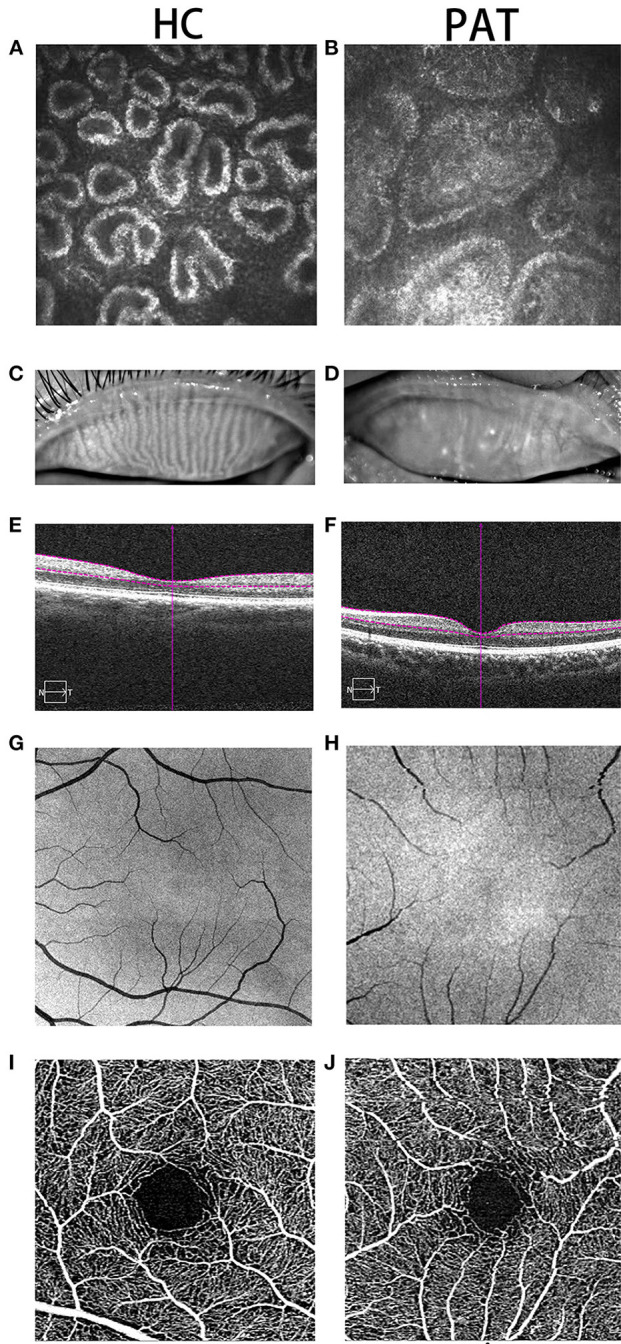
Typical pictures of HC and PAT groups. Two groups of MGs were photographed by corneal confocal microscope. As can be seen from **(A,B)**, compared with the HC group, the MG in the PAT group was significantly blocked, and gland blockage was widened at the same time. What's more, we photographed the changes of the MGs under the naked eye in the HC group and the PAT group. From **(C,D)**, we can clearly observe that the MG in the HC group is clearer than that in the PAT group. Therefore, we can speculate that obesity may lead to tarsal gland blockage and degeneration. **(E,F)** According to the cross-sectional images of retinal thickness obtained, we can observe that the choroidal thickness in the PAT group is significantly thinner than that in the HC group. We also observed that the capillaries in the HC group **(G,I)** were more developed than those in the PAT group **(H,J)** according to the fundus vascular images we obtained, which showed that obesity may reduce the thickness of choroid and fundus blood supply, and may further cause fundus lesions. HC, healthy control; PAT, MGD patient in SO population; MG, meibomian gland.

Obesity is usually accompanied by dyslipidemia, a major contributor to metabolic disease (4). Dyslipidemia is also considered to be a risk factor for MGD, which is the main cause of dry eye (5). The meibomian gland (MG) is a modified sebaceous gland in the eyelid. It produces meibomian, which is the lipid component of the tear film (6). MGD is the main cause of evaporative dry eye and is also one of the most common diseases encountered by ophthalmic nurses. MGD is characterized by obstruction of the duct at the end of the MG and/or changes in gland secretion, leading to changes in tear film stability, inflammation and irritation (7). The MG produces lipids that make up the meibomian, which have something in common with the blood. Some anecdotal reports have shown that dyslipidemia in obese patients can lead to MGD and change the composition of the meibomian lipid. Destruction of the meibomian lipid components can also increase the possibility of inflammation, an underlying cause of blepharitis, which affects the secretory function of the MG (6). This may result in decreased tear film quality and stability, and eventually leads to ocular surface inflammation in patients with MGD and dry eye (5). We observed that many SO people also had MGD. Due to the particularity of its anatomy, routine examination and diagnosis of MGD mainly relies on a corneal confocal microscope and fundus anatomy (Figure 1).

We use (fMRI) to detect brain changes (8, 9). fMRI is the mainstay of neuroimaging in cognitive neuroscience. Further development of image acquisition protocols, scanner technology, experimental design and analysis methods is expected to promote the capability of fMRI from simple mapping to actual research on brain tissues (10). fMRI can also be used to monitor the spontaneous activity of the human brain and offer a fresh explanation for the pathogeny of some diseases (11). Since it does not need any radioactive tracer, fMRI is appropriate for central mechanism studies. It can also detect and precisely position the spontaneous activity of the cerebrum via integrating functional and structural imaging (12). fALFF is an r-fMRI indicator that represents an improvement from ALFF and can more sensitively and specifically reflect regional spontaneous cerebral activity depending on blood oxygen level-dependent (BOLD) signal (13). Electrophysiologic research (14) has already indicated that low-frequency concussions are probably caused by spontaneous nerve actions, which is of great physiological importance. Further, the informational interactions between involved cerebral regions are reflected in the rhythmic activities of brain regions. fALFF enables us to identify brain areas with abnormal local functioning, thereby offering us a possible pathogenesis of MGD in SO population.

The purpose of this study was to explore the changes in spontaneous cerebral activity in MGD patients in SO population, and to explore its relationship with clinical features using fALFF.

## Participants and Methods

### Participants

A total of 12 MGD patients in SO population (8 men and 4 women, PATs) were enrolled from the Ophthalmology Department of the First Affiliated Hospital of Nanchang University. Inclusion criteria were: (1) BMI ≥ 30; (2) male' waistline ≥ 90 cm; female' waistline ≥ 85 cm; (3) diagnosed with MGD; and (4) capable of undergoing MRI scanning (no contraindications of magnetic resonance imaging such as cardiac pacemaker or implanted metal devices).

Exclusion criteria: (1) a history of a serious craniocerebral injury; (2) severe mental dysfunction; (3) history of excessive drinking; (4) history of drug abuse; (5) pregnancy; and (6) diseases that may cause changes in fMRI (systemic diseases, degenerative diseases, demyelinating diseases, and autoimmune diseases).

Twelve healthy controls (6 men and 6 women, HCs) were enlisted in this study, all of whom were age and gender matched with the PAT group. All HCs met the following criterion: (1) 24 ≥ BMI ≥ 20; (2) can undergo an MRI scan; (3) head MRI shows normal brain parenchyma; (4) no mental illness; and (5) no diseases that may cause changes in fMRI (systemic diseases, degenerative diseases, demyelinating diseases, and autoimmune diseases).

### MRI Parameters

A Trio 3-Tesla MR scanner (Siemens, Munich, Germany) was utilized for conducting the process. Each participant was asked to stay sober, make sure their eyes closed, and breathe mildly during the scanning. After that, the datum were gathered by 3D damage gradient echo sequence with the subsequent augments: for 176 configurable image scans, we used a repetition time = 1900 ms, collection matrix = 256 × 256, visual field = 250 × 250 mm, echo time = 2.26 ms, thickness = 1.0 mm, interval = 0.5 mm, rollover angle = 9°. For 240 functional image scans, we utilized a repetition time = 2000 ms, acquisition matrix = 64 × 64, field of view = 220 × 220 mm, thickness = 4.0 mm, interval = 1.2 mm, echo time = 30 ms, rollover angle = 90°, and 29 axials. Every scan continued for 15 min or so.

### FMRI Data Analysis

All functional datum was handled by a software percolator (www.MRIcro.com), and statistical parameter graphing was operated with SPM8 (The MathWorks, Inc., Natick, MA, USA) and rs-fMRI DPARSFA (http://rfmri.org/DPARSF) software data conducting coadjutants. The primary procedures of pretreatment included slice timing, head motion rectification, exerting Friston six-head movement parameters to eliminate head movement influences, dimensional standardization with normal echo complanate picture stencils to achieve Neurology Montreal Institute (MNI) standards, and smoothening with a Gaussian kernel of 6 × 6 × 6 mm3 full-width at half-maximum (FW-HM). After correcting the head movement, we applied the standard echo plane image stencil to standardize the functional image for meeting the spatial standard of the Montreal Institute of Neurology. The overall impacts of changeability were diminished by distinguishing the fALFF values of every voxel by the overall average values of every participant.

### Brain-Behavior Correlation Analysis

We utilized the resting-state fMRI datum analysis tool cabinet software; the cerebrum areas with disparate fALFF results between the two groups were divided into diverse portions. Definitively, correlative analysis was utilized to calculate the correlation between the medial fALFF values and behavior performance in every region of the PAT group (*P* < 0.05 significant differences).

### Statistical Analysis

For fMRI data, a two-sample t-test was performed using the REST toolbox to examine the voxel-wise difference between the PATs and thr HCs; State Key Laboratory of Cognitive Neuroscience and Learning, Beijing Normal University, Beijing, China (The statistical threshold was set at the voxel level with *P* < 0.05, alphasim, and cluster size > 100 voxels for multiple comparison). These voxels were considered as regions of interest with prominent differences between the two groups.

## Results

### Demographics and Behavioral Results

Age (*P* = 0.365) and gender (*P* = 0.430) were equivalent between the PATs and HCs. However, differences between the two groups were observed in their visual acuity, daily life score and mini-mental state examination (*P* < 0.05; Table 1).

**Table 1:** Basic Information of participants in the study.

**Condition**	**PAT**	**HC**	**t**	*P*-value^*^
Male/female	4/8	6/6	N/A	0.430
Age (years)	34.25 ± 7.07	31.67 ± 5.98	0.925	0.365
Weight (kg)	111.92 ± 13.33	66.08 ± 10.41	8.986	<0.01^#^
Handedness	12R	12R	N/A	>0.99
Initial visual acuity-left eye (log Mar)	0.80 ± 0.16	0.58 ± 0.09	3.742	<0.01^#^
Initial visual acuity-right eye (log Mar)	0.83 ± 0.22	0.62 ± 0.13	2.813	<0.05^*^
Daily life score	90.92 ± 6.53	100.00 ± 0.00	4.617	<0.01^#^
MMSE	21.42 ± 4.37	27.83 ± 2.41	4.266	<0.01^#^

* *P,0.05*;

# *P,0.01; independent t-test, P-values between PATs and HCs. Data presented as mean ± standard deviation. HCs, healthy controls; PATs, MGD patients in SO population; MMSE, mini-mental state examination*.

### FALFF Differences Between PATs and HCs

**Figure 2:**
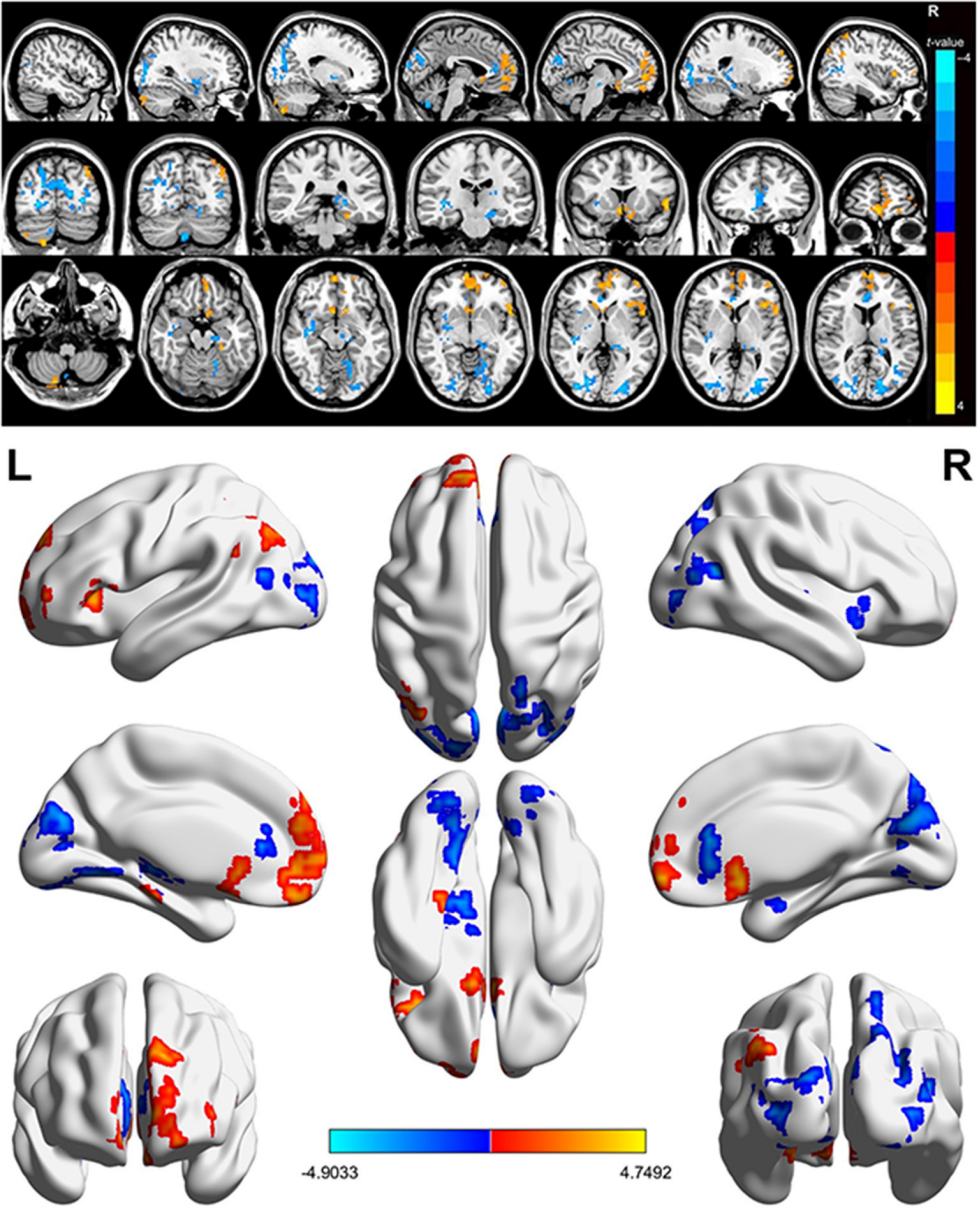
Spontaneous brain activity in MGD patients in SO population. Red regions (left lingual gyrus, right lenticular globus pallidus, right anterior cingulate and paracingulate gyrus, left middle occipital gyrus) indicate higher fALFF values, while blue regions (right cerebellum, left fusiform gyrus, right medial orbitofrontal gyrus, left triangle inferior frontal gyrus, left inferior parietal gyrus) represent lower fALFF values (*P* < 0.05; AlphaSim-corrected; cluster size, >40). SO, severe obesity; MGD, meibomian gland dysfunction; fALFF, fractional amplitude of low-frequency fluctuations.

**Figure 3:**
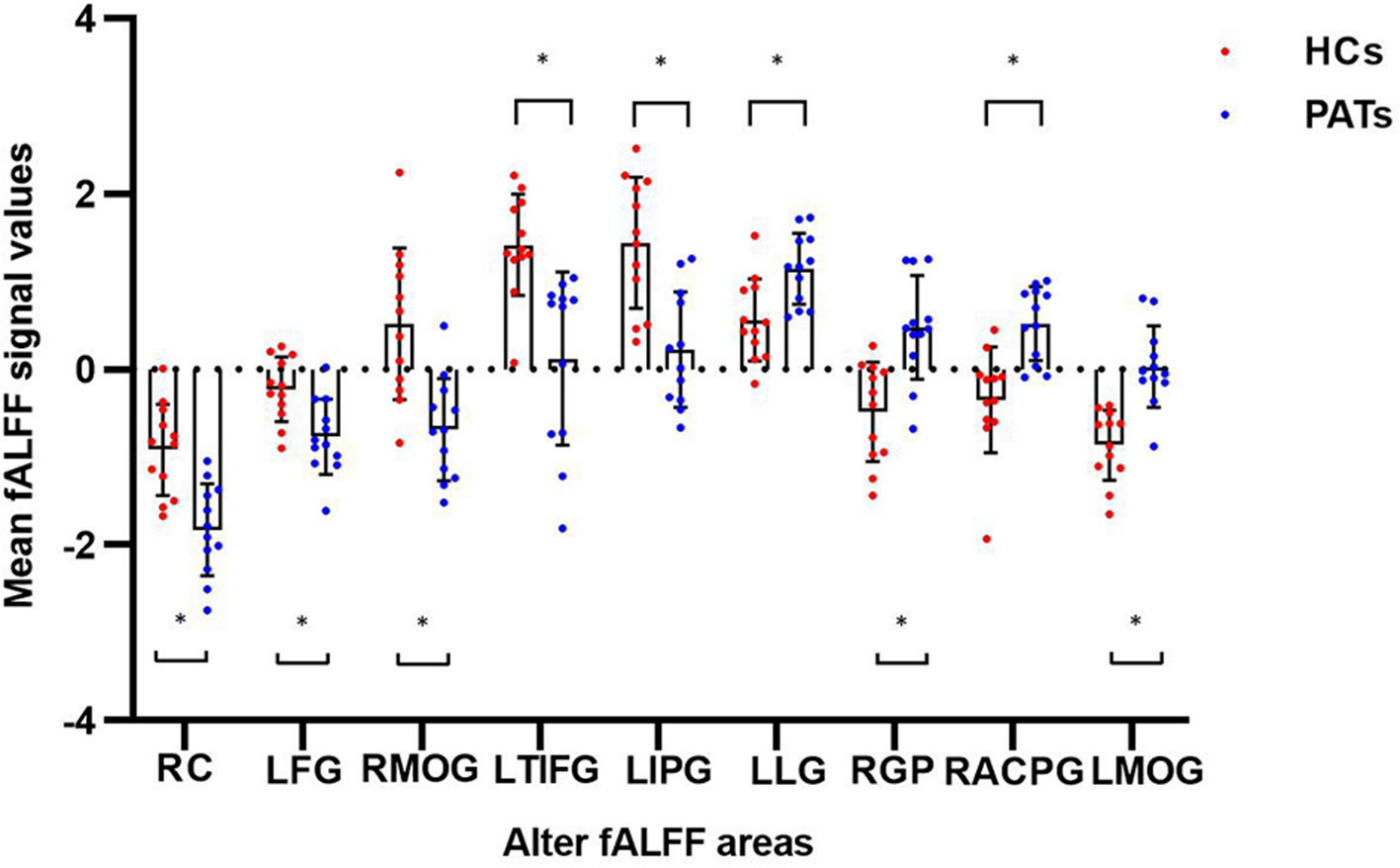
The mean fALFF values between the PATs group and HCs. Means of altered spontaneous brain activity between the PATs group and HCs group (each *n* = 12). Compared with HCs, asterisk means the statistical significance *p* < 0.05. RC, right cerebellum; LFG, left fusiform gyrus; RMOG, right medial orbitofrontal gyrus; LTIFG, left triangle inferior frontal gyrus; LIPG, left inferior parietal gyrus; LLG, left lingual gyrus; RGP, right globus pallidum; RACPG, right anterior cingulate and paracingulate gyri; LMOG, left middle occipital gyrus; fALFF, fractional amplitude of low-frequency fluctuations; HCs, healthy controls; PATs, MGD patients in SO population.

**Figure 4:**
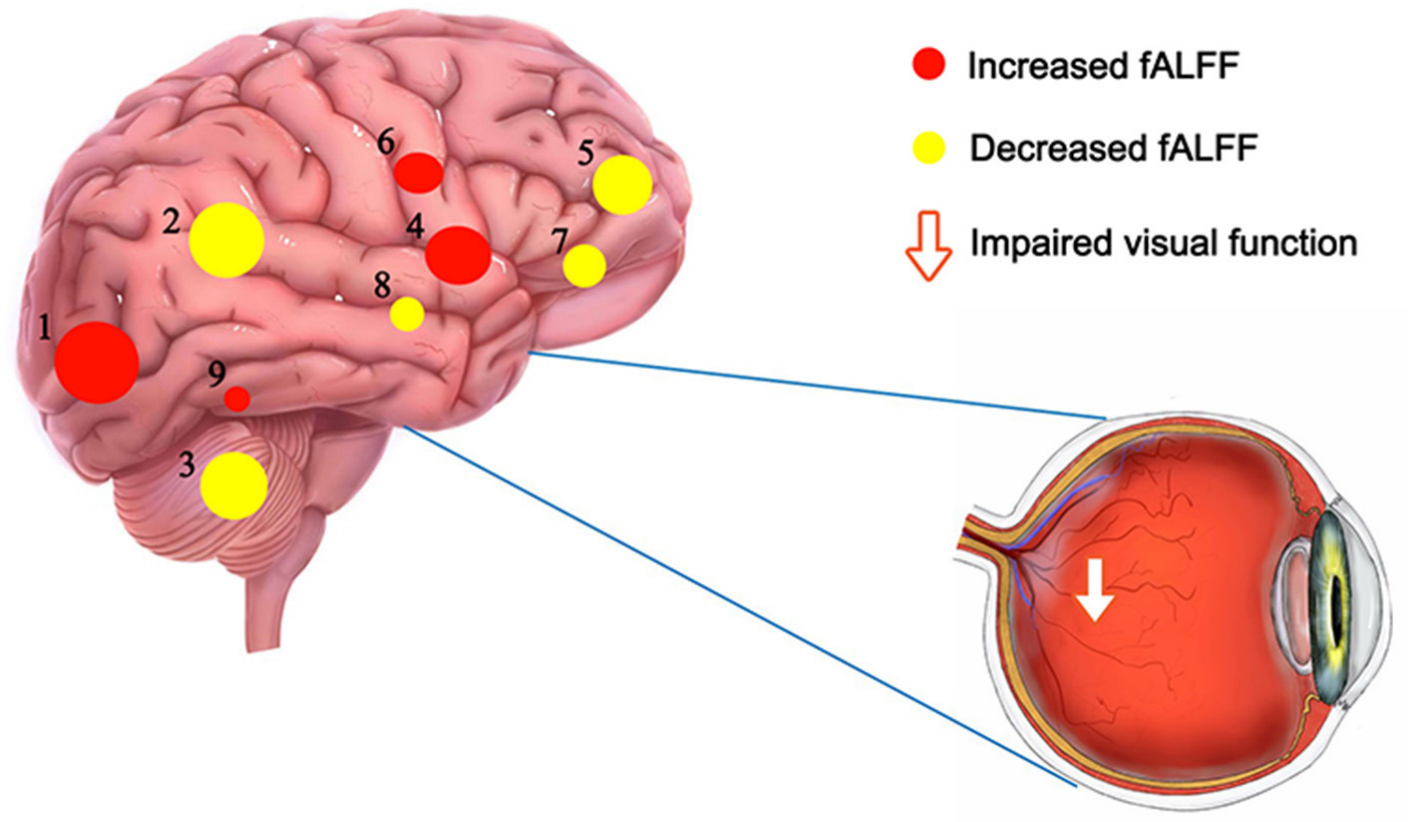
The fALFF results of brain activity in the PAT group. Compared with the HCs, the fALFF of the following regions were increased to various extents: 1-left middle occipital gyrus (t = 4.9), 4-right globus pallidum (t = 4.36), 6-right anterior cingulate and paracingulate gyri (t = 4.24), 9-left lingual gyrus (t = 3.54), and decreased fALFF values in the 2-left inferior parietal gyrus (t = – 4.75), 3-right cerebellum (t = – 4.69), 5-right medial orbitofrontal gyrus (t = – 4.35), 7- left triangle inferior frontal gyrus (t = – 4.05) and 8-left fusiform gyrus (t = – 3.688). The sizes of the spots denote the degree of quantitative changes. HCs, healthy controls; fALFF, fractional amplitude of low-frequency fluctuations; PAT, MGD patient in SO population.

In contrast with HCs, PATs had significantly decreased fALFF values in the right cerebellum, the left fusiform gyrus, the right medial orbitofrontal gyrus, the left triangle inferior frontal gyrus and the left inferior parietal gyrus, which means the activity of those brain regions are reduced, indicating dysfunctional brain activity in those lobes. Further, PATs had increased fALFF values in the left lingual gyrus, the right globus pallidus, the right anterior cingulate and paracingulate gyri and the left middle occipital gyrus, which means these brain regions are more active than the normal, indicating that there might be compensatory processes for some impaired function in other brain regions (Figures 2–4 and Table 2).

**Table 2:** Brain regions with significant differences in fALFF between the HC and PAT groups.

**fALFF**	**Brain areas**	**MNI coordinates**			
		**X**	**Y**	**Z**	**BA**	**Peak voxels**	***T*-value**
HC > PAT							
1	RC	21	−81	−57		152	4.69
2	LFG	−27	−33	−18	36	103	3.688
3	RMOG	3	60	−12	10	440	4.35
4	LTIFG	−54	15	−6	45	100	4.05
5	LIPG	−48	−63	51	40	116	4.75
HC < PAT							
6	LLG	−18	−69	−12	19	106	−3.54
7	RGP	39	−21	0	13	115	−4.36
8	RACPG	6	33	12	24	138	−4.24
9	LMOG	−30	−78	3	18	782	−4.9

*For fALFF data, two-sample t-test was performed to examine the voxel-wise difference between the PAT and HC groups using the REST toolbox. The statistical threshold was set at the voxel level with P < 0.05, FDR corrected, and cluster size>100 voxels for multiple comparison. These voxels were regarded as the regions of interest showing significant difference between the two groups.fALFF, fractional amplitude of low-frequency fluctuations; HC, healthy control; PAT, MGD patient in SO population; RC, right cerebellum; LFG, left fusiform gyrus; RMOG, right medial orbitofrontal gyrus; LTIFG, left triangle inferior frontal gyrus; LIPG, left inferior parietal gyrus; LLG, left lingual gyrus; RGP, right globus pallidum; RACPG, right anterior cingulate and paracingulate gyri; LMOG, left middle occipital gyrus*.

### Receiver Operating Characteristic Curve

**Figure 5:**
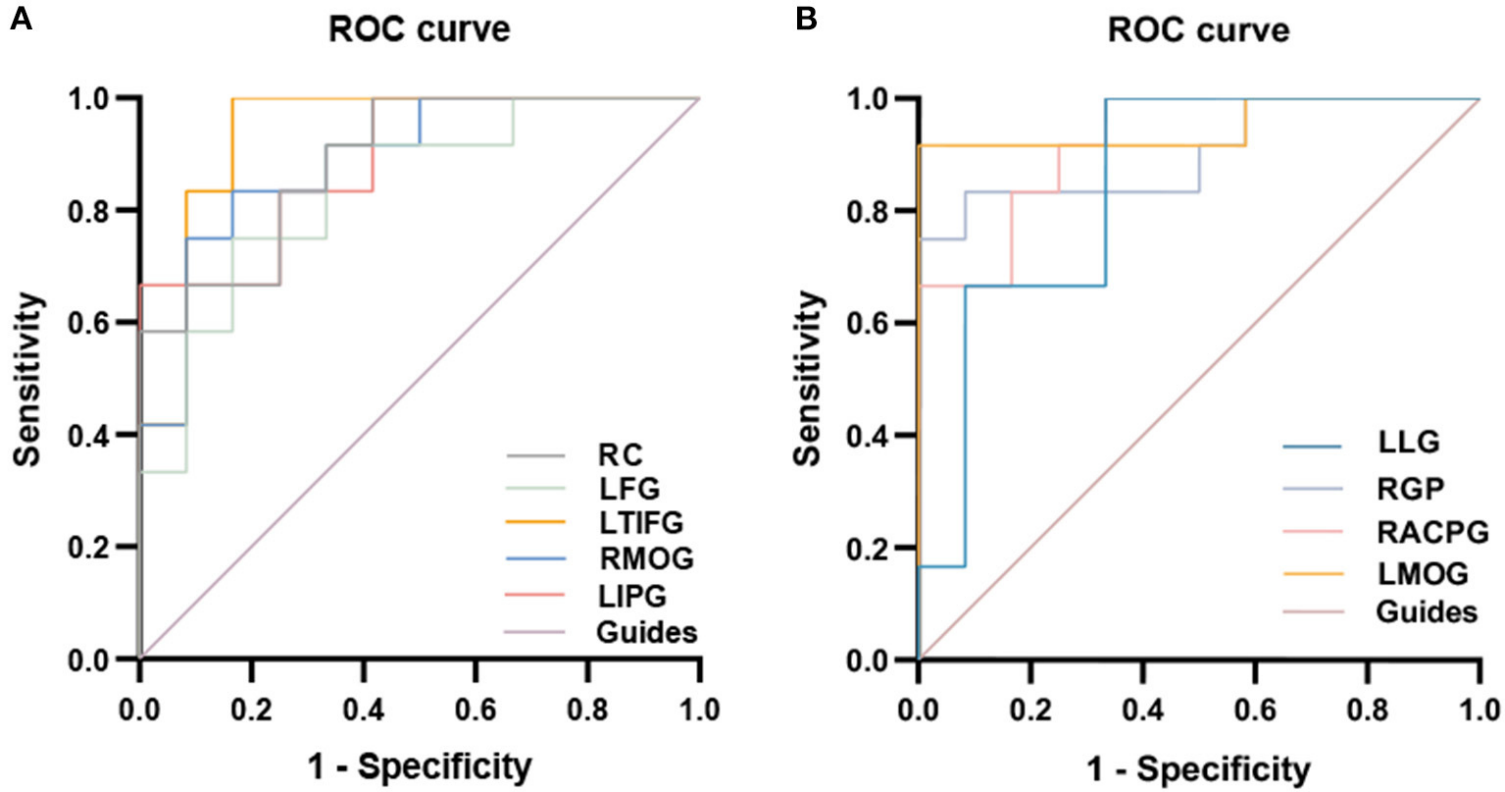
ROC curve analysis of the mean fALFF values for altered brain regions. **(A)** The area under the ROC curve were 0.8889 (*p* < 0.005; 95% CI 0.7620–1.000) for RC, LFG 0.8403 (*p* < 0.005; 95% CI 0.6802–1.000), RMOG 0.8889 (*p* < 0.005; 95% CI 0.7580–1.000), LTIFG 0.9375 (*p* < 0.001; 95% CI 0.8351–1.000), LIPG 0.8889 (*p* < 0.005; 95% CI 0.7606–1.000) [HCs > PATs]. **(B)** The area under the ROC curve were 0.8472 (*p* < 0.005; 95% CI 0.6836–1.000) for LLG, RGP 0.9028 (*p* < 0.001; 95% CI 0.7744–0.992), RACPG 0.9236 (*p* < 0.001; 95% CI 0.8219–1.000), LMOG 0.9514 (*p* < 0.001; 95% CI 0.8547–1.000) [HCs < PATs]. fALFF, fractional amplitude of low-frequency fluctuations; ROC, receiver operating characteristic; RC, right cerebellum; LFG, left fusiform gyrus; RMOG, right medial orbitofrontal gyrus; LTIFG, left triangle inferior frontal gyrus; LIPG, left inferior parietal gyrus; LLG, left lingual gyrus; RGP, right globus pallidum; RACPG, right anterior cingulate and paracingulate gyri; LMOG, left middle occipital gyrus; HCs, healthy controls; PATs, MGD patients in SO population.

There were significant differences in the fALFF values between PATs and HCs. We therefore decided that fALFF values could be used to differentiate PATs from HC. We then drew a ROC curve to analyze the medial fALFF values of disparate cerebral areas. The area under the curve (AUC) denoted the diagnosis rate. A value of 0.5 to 0.7 signified low accuracy, 0.7 to 0.9 signified medium accuracy, and >0.9 signified high accuracy. The AUCs were 0.8889 (*p* < 0.005; 95% CI: 0.7620–1.000) for the right cerebellum (RC), 0.8403 (*p* < 0.005; 95% CI: 0.6802–1.000) for the left fusiform gyrus (LFG), 0.8889 (*p* < 0.005; 95% CI: 0.7580–1.000) for the right medial orbitofrontal gyrus (RMOG), 0.9375 (*p* < 0.001; 95% CI: 0.8351–1.000) for the left triangle inferior frontal gyrus (LTIFG) and 0.8889 (*p* < 0.005; 95% CI: 0.7606–1.000) for the left inferior parietal gyrus (LIPG) [HCs > PATs]. The AUCs were 0.8472 (*p* < 0.005; 95% CI: 0.6836–1.000) for the left lingual gyrus (LLG), 0.9028 (*p* < 0.001; 95% CI: 0.7744–0.992) for the right globus pallidum (RGP), 0.9236 (*p* < 0.001; 95% CI: 0.8219–1.000) for the right anterior cingulate and paracingulate gyri (RACPG), and 0.9514 (*p* < 0.001; 95% CI: 0.8547–1.000) for the left middle occipital gyrus (LMOG) [HCs < PATs]. In summary, these results indicate that the fALFF values of different areas of the cerebral areas might help in the diagnosis of MGD in SO patients. In addition, the ROC curves showed that the fALFF values of LTIFG, RACPG, LMOG, RC, RMOG, LIPG and RGP were more clinically relevant than LFG and LLG (Figure 5).

### Rs-FMRI-FALFF Value of PATs' Brain Regions and HADS Scores

**Figure 6:**
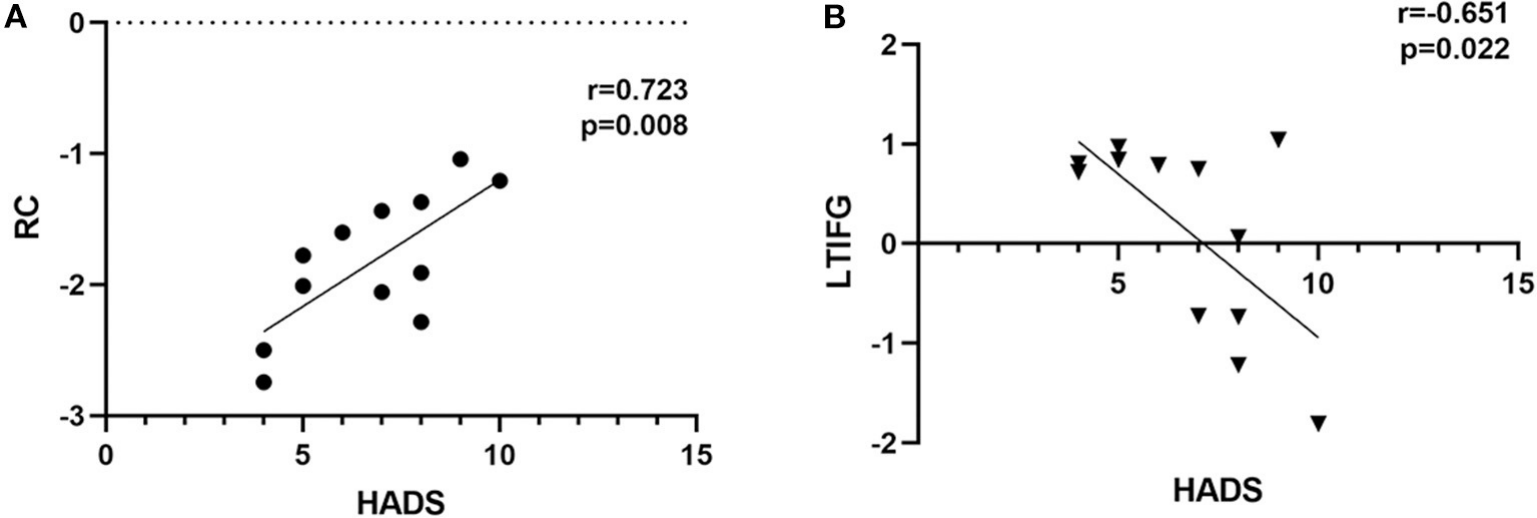
Correlation analysis result. **(A)** The fALFF values in the right cerebellum of PAT presented significantly positive correlation with HADS (r = 0.723, *P* = 0.008). **(B)** The fALFF values in the left triangle inferior frontal gyrus of PAT presented significantly negative correlation with HADS (r = −0.651, *P* = 0.022). fALFF, fractional amplitude of low-frequency fluctuations; HADS, hospital anxiety and depression scores; PAT, MGD patient in SO population.

The fALFF values of the right cerebellum of PATs had a significantly positive correlation with HADS (r = 0.723, *P* = 0.008), and the fALFF values of the left triangle inferior frontal gyrus of PATs were significantly negatively correlated with HADS (r = −0.651, *P* = 0.022) (Figure 6).

## Discussion

Obesity is related to severe health risks (15). Severe obesity further increases the risk of obesity-correlative complications such as coronary heart disease, end-stage kidney disease and MGD. This study was designed to investigate the connection between MGD patients in SO population and functional cerebral changes using the fALFF technique. In contrast with HCs, PATs had significantly decreased fALFF values in their right cerebellum, left fusiform gyrus, right medial orbitofrontal gyrus, left triangle inferior frontal gyrus and left inferior parietal gyrus. In contrast, increased fALFF values were seen in PATs in the left lingual gyrus, the right globus pallidus, the right anterior cingulate and paracingulate gyri and the left middle occipital lobe. fALFF has been used in a variety of diseases with success and its clinical use is likely to expand (Table 3). We did some research on brain regions alternations and its potential impact to better understand the brain changes in the PATs (Table 4).

**Table 3:** fALFF method applied in systemic, neurogenic and mental diseases.

	**Author**	**Year**	**Disease**
Systemic	Zhang et al. (16)	2021	Subclinical Hypothyroidism
diseases	Quan et al. (17)	2022	Acute Basal Ganglia Ischemic Stroke
	Zhang et al. (18)	2021	neovascular glaucoma
	Bak et al. (19)	2018	AIDS
	Zhang et al. (20)	2019	Diabetes
Neurogenic	Rong et al. (21)	2021	Parkinson's Disease
diseases	Luo et al. (22)	2020	Hemifacial Spasm
	Fulong et al. (23)	2018	Narcolepsy with Cataplexy
	Kim et al. (24)	2021	Migraine
Mental	Qiu et al. (25)	2019	Major depressive disorder
diseases	Egorova et al. (26)	2017	Post-stroke depression
	Xu et al. (27)	2015	Schizophrenia
	Qiu et al. (28)	2021	Bipolar disorder
	Liu et al. (29)	2021	Cognitive Impairment

*fALFF, fractional amplitude of low-frequency fluctuations*.

**Table 4:** Brain regions alternations and its potential impact.

**Brain regions**	**Experimental result**	**Brain function**	**Anticipated results**
Right cerebellum	HCs > PATs	Language and executive functions, physical balance, motor coordination,	AD,depression, Schizophrenia
Left fusiform gyrus	HCs > PATs	Visual word formation, faces and words processing	Dyslexia, prosopagnosia, alexia
Right medial orbitofrontal gyrus	HCs > PATs	Social evaluation, decision making, affective representation	anxiety, PTSD, Catatonia, Schizophrenia
Left triangle inferior frontal gyrus	HCs > PATs	Searching space extension, concepts selection, information evaluation, association inhibition	Depression, anorexia nervosa, dyslexia, conversion disorder
Left lingual gyrus	HCs < PATs	dreaming, facial expressions, terrain, face and visual word recognition	CUQ, hyperactivity with “visual snow”
Right globus pallidum	HCs < PATs	Learning and adjustment, development and control of habitual behaviors	Low self-efficacy, Huntington's disease
Right anterior cingulate and paracingulate gyri	HCs < PATs	Emotional regulation, cognitive processes, and specific motor functions.	AD,TAO,BVFD
Left middle occipital gyrus	HCs < PATs	Language, concept, number, analysis, logical reasoning, visual processing, perception	Major depression, high myopia

*HCs, healthy controls; PATs, MGD patients in SO population; AD, Alzheimer's disease; PTSD, post-traumatic stress disorder; TAO, thyroid-associated ophthalmopathy; BVFD, behavioral variant frontotemporal dementia; CUQ, contralateral upper quadrantanopsias*.

From the results, we can see that the PATs have significantly decreased fALFF values in the right cerebellum. The right cerebellum plays a critical role in the collaboration between reflex and autonomic motion. An increasing amount of evidence has shown that the right cerebellum also plays a role in cognition and emotion (30). In late-onset depression patients, excessive cerebellar functional connectivity (FC) with the medial prefrontal lobe was significantly associated with depression symptoms (31). Su et al. (32) studied the cerebral metabolism of patients with depression using PET, suggesting that changes in cerebellar metabolism likely play an important role in depression pathophysiology. Several metabolic-related studies have found that the metabolic activity of the right posterior cerebellar lobe in patients with major depression is enhanced (33). Previous studies have also reported reduced cerebral blood flow in the cerebellums of depression patients (34). Furthermore there's research suggested that the functional decline in cerebellar gray matter is related to Alzheimer's disease (AD) (35). Other studies have shown that interruption of the connection between the cerebellum and the right dorsolateral prefrontal cortex is related to the severity of negative symptoms, leading to schizophrenia (36). These studies show a potential relation of the PATs with cognition and emotion diseases such as depression, AD and schizophrenia. This goes in line with our findings that the fALFF values of the right cerebellum of PATs had a significantly positive correlation with HADS.

The fusiform gyrus (FG), also called the lateral occipitotemporal gyrus, is a portion of the temporal lobe and occipital lobe in Brodmann area 37. Although the function of the FG is not completely clear, it has been connected with a large number of neural pathways associated with recognition. Moreover, the FG has been connected with a variety of neurological diseases such as dyslexia, prosopagnosia and alexia. The visual form area of the left FG is usually in a low activation state in dyslexia patients (37, 38). There is increasing evidence that the FG is involved in face and word processing. Selective damage to the FG can lead to serious defects in facial recognition, but printed character recognition remains relatively intact (39). There are different facial and word selection areas in the FG (40). It is conceivable that lesions that only destroy the areas of the FG that are selective to the face will lead to relatively simple facial agnosia, while lesions in the areas of the FG that are selective to words will lead to relatively simple alexia. In the present study, we found that the PATs showed reduced fALFF values in the left FG, and this might be reflective of dysfunction in that region, which means the PATs may have potential relations with the neurological diseases mentioned above.

The right medial orbitofrontal cortex (mOFC) is a critical area that records various high-order motor control processes (41). Changes in the mOFC may lead to a variety of neuropsychiatric disorders and their clinical symptoms (42). Function of the frontal limbic areas, especially the mOFC, is related to social evaluation, decision-making and emotional representation (43), and its impairment may directly affect the social driving force and emotion of patients with negative symptoms. de Leeuw et al. (44) observed that abnormal reward-related activation in the striatum was associated with negative symptoms in patients with schizophrenia compared with their unaffected siblings. Shukla et al. (45) also reported a decrease in the functional connectedness of the frontal striatum of patients with schizophrenia, and suggested that there is a connection between the striatum and the right mOFC in the development of negative symptoms. Hirjak et al. (46) confirmed that schizophrenic spectrum disorder (SSD) patients with catatonia mainly showed a reduction in the surface area of the mOFC compared with non-catatonic patients. mOFC anomalies have also been detected in a variety of anxiety disorders (47). Structural MRI studies suggested that reduced mOFC volume was related to anxiety disorder. Previous study found that cancer survivors with post-traumatic stress disorder (PTSD) had reduced OFC volume compared with cancer survivors and healthy groups without PTSD (48). In the present study, we found that the PATs exhibited lower fALFF values in the right medial orbitofrontal gyrus, indicating the dysfunction of them, which may be the reason for emotional disorders in the PATs.

The left triangle inferior frontal gyrus is a section of the inferior frontal gyrus (IFG) that is situated between the ascendant branch and the anterior branch. It is the central part of Broca's athletic language region and is involved in sensory and emotional information evaluation (49). Becker et al. (50) summarized that the IFG, which is already active during search and solution in verbal creative problem solving, is likely to take part in the extension of the search space that leads to the right solution. In addition, the IFG pars triangularis likely participates in the inhibition of solution irrelevant associations. The IFG pars triangularis is also associated with concept selection by means of solving competitions between active representations (51). Cheng et al. (52) reported that there is some connection between the IFG pars triangularis and Parkinson's syndrome. Qi et al. (53) pointed out that children's language performance is related to the asymmetric changes of cortical thickness in the IFG pars triangularis. In addition, Yang et al. (54) further found that the faster execution control response time was related to the smaller gFCD value in the trigonum of the IFG. In the this study, we found that the PATs exhibited lower fALFF values in the left triangle inferior frontal gyrus, indicating dysfunctional brain activity in those lobes, which may lead to emotional disorders in the PATs.

The lingual gyrus (LG) adjoins the continuous parahippocampal gyrus in the front, connecting to the talar sulcus on the medial side and the accessory sulcus separated from the medial inferior fusiform gyrus on the lateral side. It is related to terrain recognition, face recognition and dreaming. Bilateral LG activation is related to facial expression. The LG is also related to the visual recognition of characters. Lesions in the LG are related to contralateral upper quadrant insomnia and hyperactivity with “visual snow” (55). Meadows et al. (56) stated that a LG lesion was involved in prosopagnosia. Areas of abnormal brain color vision were also found in the occipitotemporal lobe (57), more precisely in the LG and the fusiform gyrus (58, 59). Bilateral lesions of the lingual and fusiform gyri can lead to achromatopsia, while unilateral right- or left-sided lesions may result in hemiachromatopsia (60). We found that the PATs have significantly higher fALFF values in the left LG, which may indicate the functional improvement to compensate for impaired visual acuity.

The globus pallidum (GP) is the main striatal outflow target and a central structure of the basal ganglia. It is a triangular cell mass located inside the putamen (61). Neuroimaging studies (62–64) have shown that patients with Huntington's disease (HD) have severe atrophy of the GP. A brain structure study on factors related to general self-efficacy in young people reported that the degree of motivation is related to an above average diffusion rate of the right putamen, GP and caudate nucleus, and the degree of physical activity is related to the right putamen (65). Nakagawa et al. (66) reported that self-efficacy scores on the General Self-Efficacy Scale were related to lower mean diffusivity values in the lenticular nucleus (putamen and GP). GP also seems to be related to learning and adaptation. The dorsolateral posterior putamen/GP area likely plays a crucial role in the development and management of human habitual manifestation (learning) (67). Furthermore, the inner part of the GP projects to the thalamus and brainstem nucleus, which control motor behavior (regulation) (68). Insufficient function of the lenticular nucleus may therefore lead to decreased self-efficacy. Volume reduction in the indirect pathway involving the dorsolateral side of the right GP is related to the reduced understanding of the causal effects of goal-directed behavior in young patients with depression (66). In the present study, we found that the PATs exhibited significantly higher fALFF values in the right GP, which may be a compensatory process of emotional disorders.

A large number of neuroimaging studies have suggested that the anterior cingulate cortex is related to cognitive processes, emotional regulation and specific motor functions (69). Silkiss et al. (70) found that the gray matter layer of the right anterior cingulate gyrus became thinner and linked this to the emotional manifestations of thyroid-associated ophthalmopathy. Feng et al. (71) discovered that patients with Alzheimer's Disease had FC destruction in certain brain areas of the left hippocampal functional network, which includes the right anterior cingulate gyrus and the paracingulate gyrus. Moreover, the FC was reduced in certain brain areas of the right hippocampal functional network, including the bilateral anterior cingulate gyrus (71). Prior imaging (72) and pathologic case series (73) have focused on the regional relevance of the anterior cingulate, orbitofrontal and anterior insular cortices. The right hemisphere tends to contribute to behavioral variant frontotemporal dementia. In the present study, we found that the PATs have significantly higher fALFF values in the right anterior cingulate and paracingulate gyrus, which may reflects a compensatory process of emotional disorders.

The left middle occipital gyrus (L-MOG), which lies in the left hemisphere, takes part in a variety of functions such as analysis, language, notion, digit and logic. The occipital gyrus is associated with visual processing, especially early visual processing (74). MOG is also involved in the perception of softness (75), and its response to texture is more sensitive than proprioception (76). The occipital lobe, which includes the bulk of the optical cortex, is involved in communication with the cerebral cortex and the process of visual information. It also participates in the perception of facial emotion. Teng et al. (77) discovered that the fALFF of the left middle occipital gyrus was observably lower in patients with major depression than in healthy controls. An additional study characterized the role of the MOG in the regulation of unconscious face/tool processing by category-selective attention, and found that the activation of the MOG decreased under facial selective attention in the course of unconscious face processing (78). In accordance with these studies, fALFF of the L-MOG likely offers a neural basis for the interruption of visual processing in female patients with major depression. Further, high myopia patients exhibited reduced cortical surface thickness in the L-MOG compared with HCs. Wu et al. (79) believed that the multi-layer cortical thickness of patients with high myopia (HM) changed, suggesting that HM may lead to structural changes in relevant brain areas, in fragile areas that result in graver deterioration of myopia or both (79). We found that the PATs showed higher fALFF values in the L-MOG, which may be a compensatory process of impaired visual acuity and emotional disorders.

**Figure 7:**
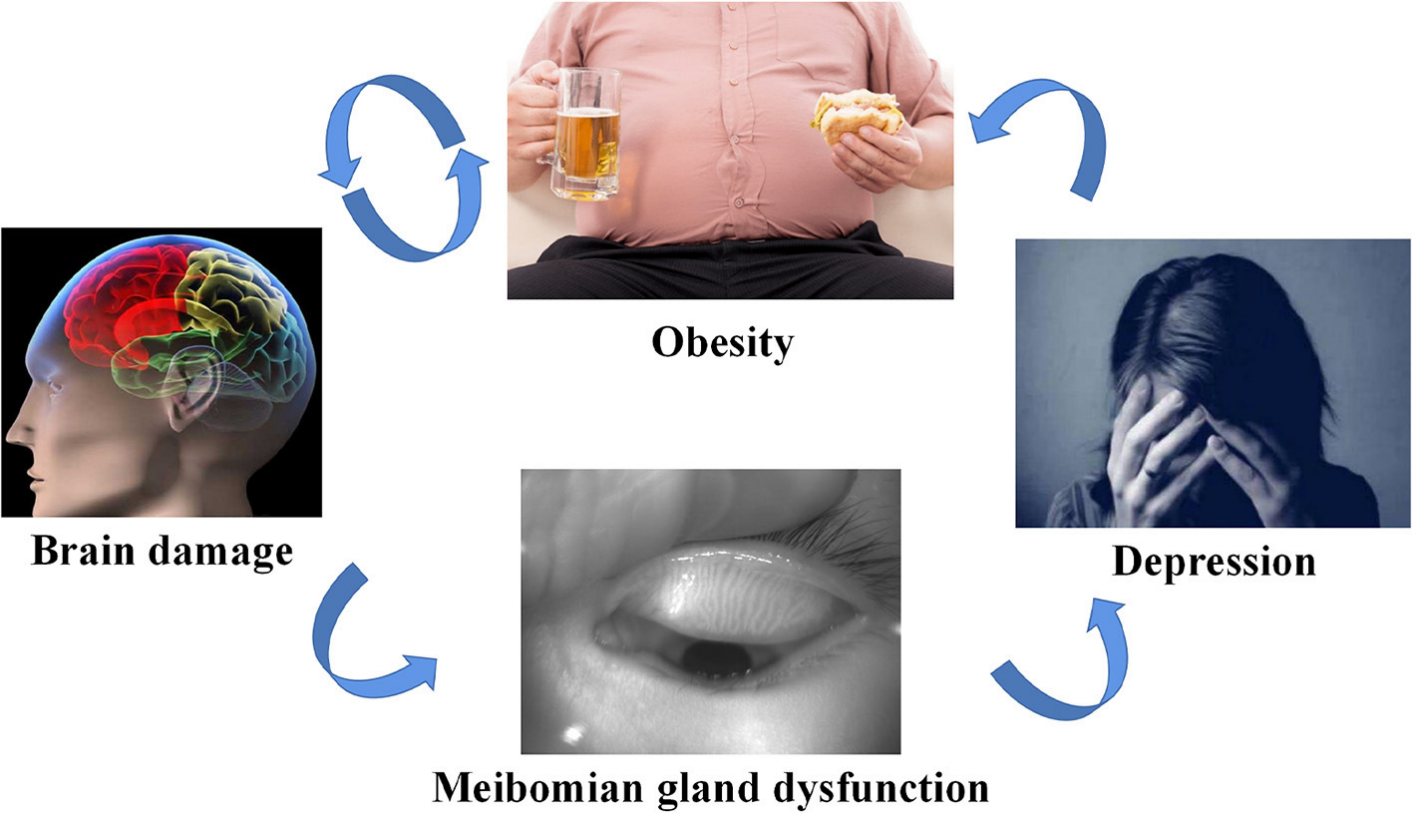
Schematic diagram of the relationship between SO, brain dmage, MGD and depression. Compared with the HCs, fALFF values of the right cerebellum were significantly decreased in the PATs, which were more likely to suffer from anxiety and depression. (Anxiety and depression are not critical symptoms of MGD in SO population). SO, severe obesity; MGD, meibomian gland dysfunction; fALFF, fractional amplitude of low-frequency fluctuations; HCs, healthy controls; PATs, MGD patients in SO population.

Our outcomes indicated that the anxiety and depression scores of the PAT group were higher than those of the HC group. It is likely that altered spontaneous brain activity is related to changes in the emotional processing of MGD patients in SO population (Figure 7).

There was limitation in our study. Our sample size is relatively small. In the follow-up in-depth study, we will expand the sample size to exclude the influence of regional and other environmental factors on the experimental results, to obtain more accurate data. But this study can still suggest some clinical significance.

## Conclusion

Our outcomes indicate that spontaneous activity alternations were detected in many areas of the cerebrum in MGD patients in SO population compared to the healthy. Abnormal fALFF values of these cerebral regions may indicate the early stage of MGD in SO population and can be used to predict and prevent the development of the disease. This research provides brand-new insights into the cerebral abnormalities of MGD in SO population from the standpoint of dynamic regional cerebral activity, emphasizing the importance of fALFF variations in clarifying the neuropathological mechanisms of MGD in SO population and may provide a method for diagnosing this disease.

## Data Availability Statement

The original contributions presented in the study are included in the article/supplementary files, further inquiries can be directed to the corresponding authors.

## Ethics Statement

The studies involving human participants were reviewed and approved by the Medical Ethics Committee of the First Affiliated Hospital of Nanchang University (Nanchang, China). The patients/participants provided their written informed consent to participate in this study.

## Author Contributions

All authors listed have made a substantial, direct, and intellectual contribution to the work and approved it for publication.

## Funding

National Natural Science Foundation (No. 82160195); Central Government Guides Local Science and Technology Development Foundation (No. 20211ZDG02003); Key Research Foundation of Jiangxi Province (Nos. 20181BBG70004 and 20203BBG73059); Excellent Talents Development Project of Jiangxi Province (No. 20192BCBL23020); Natural Science Foundation of Jiangxi Province (No. 20181BAB205034).

## Conflict of Interest

The authors declare that the research was conducted in the absence of any commercial or financial relationships that could be construed as a potential conflict of interest.

## Publisher's Note

All claims expressed in this article are solely those of the authors and do not necessarily represent those of their affiliated organizations, or those of the publisher, the editors and the reviewers. Any product that may be evaluated in this article, or claim that may be made by its manufacturer, is not guaranteed or endorsed by the publisher.
